# Hygiène Militaire, à l'usage des Armées de Terre

**Published:** 1845-04

**Authors:** 


### Art. IV.

1. *Hygiène Militaire, à l'usage des Armées de Terre.* Par le Chevalier J. R. Louis de Kirckhoff, &c. &c. 2de Edition.—*Anvers,* 1823. 1 vol. 8vo, pp. 250.
2. *Vade-mecum, ou Guide du Chirurgien Militaire.* Par le Chevalier Sarlandiere, d.m. &c. &c.—*Paris,* 1823. 1 vol. pp. 362.
3. *Manuel portatif des Officiers de Santé des Hôpitaux et des Corps de Troupes.* Par A. Dorat.—*Paris,* 1834. 1 vol. 32mo.
4. *Manuel réglementaire à l'usage des Officiers de Santé des Hôpitaux Militaires et des Corps de troupes.* Par J. A. A. E. Puel, d.m. &c. &c.—*Metz,* 1837. 1 vol. 8vo, pp. 508.
5. *Aide-memoire Medico-legal de l'Officier de Santé de l'Armée de Terre; publié avec autorisation du Ministre de la Guerre, et encouragé par le conseil de Santé des Armées.* Par F. C. Maillot, Professeur à l'hôpital militaire d'instruction de Metz; et J. A. A. E. Puel, Médecin ordinaire, &c.—*Paris,* 1842. 1 vol. 8vo, pp. 644.
6. *Elements d'Hygiène Militaire.* Par D. Ph. Mutel, d.m. Chirurgien Major au 52e de ligne, &c. &c.—*Paris,* 1843. 1 vol. 8vo, pp. 392.
7. *On Military Hygiene, and particularly upon the Clothing of Soldiers.* By Frederic Roberts, Esq. Assistant Surgeon, 59th Regiment. *Reprinted from the Medical Gazette,* pp. 24.

A remarkable feature in the literature of the present day is the number of handbooks and guides to every kind of knowledge which are almost hourly ushered into existence. We have them of all descriptions, and relating to all manner of places, from Handbooks for India, which promises by and bye to be an agreeable tour for lawyers during the long vacation, to Guides for holiday parties to Hampton Court or Gravesend. In the fine arts we have them of all sizes, from Mrs. Jameson's excellent Guide to the Public Galleries, down to Punch's amusing one to the Chinese Exhibition; we have them on almost every conceivable object,—Cookery and Life Assurance, Swimming and the Toilet, Horsemanship and Swindling, *cum multis aliis.* In most instances the supply has doubtless been created by the demand, and it may be fairly assumed from their abundance, that the want of some such works has been generally felt and their utility appreciated. A system which has been found to take so well in matters of taste, of luxury, and of comfort might be extended with advantage to the everyday duties of life, and would greatly facilitate the acquirement of those details which, in every business and profession, though trifling in themselves, tend very materially to the comfort and efficiency of the individual. There is perhaps no class to whom such a work would be more useful than to medical officers upon their first appointment to commissions in the army and navy. They must have previously gone through a course of study, in every respect sufficiently extensive, and calculated to fit them for the discharge of their professional duties in civil life, and we are entitled to infer from the amount of clinical instruction demanded of them, that most if not all are *practically* acquainted with their profession. Yet the duties they are called on to perform in the army and navy are in many respects widely different from those of a clerk in a civil infirmary, or a pupil in a dispensary. Their social position also is so altered from that they have left, whether they have been domesticated with their families or have prosecuted their studies away from home, and been already thrown on their own resources among strangers, that a work of this nature, if judiciously executed, could not fail to prove highly useful. We believe there are few medical officers who, on looking back to the early part of their career, will not agree with the following sentiments of Sarlandière: "When I joined the army, rich in professional knowledge, but very inexperienced in all that concerned the soldier, how often would a guide book have saved me from the mistakes and ignorance very natural to a man unaccustomed to live among soldiers! and how much better could I have given that information which my chiefs had a right to expect from me!" This, as well as many of the observations contained in the following pages, is equally applicable to the medical officers of the navy. In the present article, however, as the books before us all relate especially to the army, we shall keep mainly in view that department of the public service.

At present, persons who are about to be recommended for appointments in the Army Medical Department are sent to the General Hospital at Fort Pitt, for the purpose of making themselves acquainted with their future duties, so far as they can be acquired in that establishment. They are detained there, living in lodgings at their own expense, without receiving pay, or sharing any of the privileges enjoyed by officers holding Her Majesty's commission, for a period varying from a few weeks to several months, according as vacancies chance to occur. Much benefit must doubtless be derived from the facilities afforded by the magnificent museum at Chatham, of tracing the effects of disease as it occurs in tropical climates, and from the frequent opportunities which present themselves for the study of pathological anatomy, particularly at those seasons when large batches of invalids arrive from foreign stations, too often in the last stage of disease, and in a condition which preclude the possibility of their being discharged, to spend the closing days of their existence among their friends. But these advantages might be very much increased, were the principal medical officer directed to deliver clinical lectures on the numerous interesting cases which must constantly present themselves, and to instruct the candidates and junior officers in the various duties required of them both in regiments and on the staff, with occasional examinations on these subjects. "It is not in the medical schools," says Kirckhoff, "that we can make good medical officers but in the military hospitals; it is only by practising in the midst of camps and armies, and by studying the physical and moral condition of the soldiers, that the medical officer can acquire the knowledge which his duties demand—duties very different from those of the physician and surgeon in civil life." The inspectors and deputy inspectors of the Royal Naval hospitals at Haslar and Plymouth deliver, during the summer months, a course of lectures to the medical officers serving in the ships of war in port, and in the hospitals. Were a similar arrangement adopted by the staff of the general hospitals at Chatham and Cork, and there is no reason why in the army it should be confined to the summer months, there cannot be the least doubt that it would be productive of great benefit. Young medical officers would thus be instructed in their duties, and in the principles of military hygiene by men who, from their standing in the service, would be well qualified for the task; for it must be obvious that no one can convey the requisite information on these vitally important subjects, but a medical officer of long and varied experience, one who from having lived among soldiers, is practically acquainted with their habits, and has served with them in the various climates, and under the diversified conditions incident to a military life. Habits of reading and thinking would be engendered among the officers from the very commencement of their career, and they would be thus induced to store their minds with useful information, and to take advantage of the opportunities afforded them of cultivating science, promoting the interests of the service, and applying to practical purposes the theoretical knowledge they had acquired in the schools.

In France the medical officers of the army are educated at the public expense, and the course of instruction is regulated strictly with a view to their ultimate destination. There are three schools for this purpose, namely, at Strasburg, Metz, and Lille, each connected with a large military hospital, and the professors are elected by *concours* from among the medical officers of the army. After having finished in one of these schools the prescribed course of medical education, including lectures on military hygiene, and having passed a rigid examination, the pupils are sent for a year to the "hôpital de perfectionnement," at Paris. Being thus trained from the very commencement of their studies, in military hospitals, and instructed by experienced medical officers, they attain a knowledge of the moral and physical condition and of the habits of soldiers, which enables them to benefit by the prelections delivered to them, and to become well versed in the various duties they may be afterwards called on to perform.

"Of all the branches of medicine," asks Revolat, "ought that to be the least known which relates particularly to the military profession?" We see no reason why it should be so; we fear, however, that the charge of negligence in this particular may be too truly applied to our own army in the present day. Although from their education, which throughout is a practical training for a military career, the medical officers of the French army would seem to require the assistance of guide books less than the British, yet they are much better supplied with them; indeed a good work of this nature is still a desideratum in our service. It may certainly well excite surprise considering the great care that has been taken in the admission of efficient medical officers into the army during a period of thirty years, that no complete treatise on military hygiene has yet made its appearance, and that so very few standard medical works have emanated from the officers of this department.

We have noted a few of the more recent French publications at the head of this article, but had we extended our investigations a little further back, and included works which from their size cannot be termed "hand-books," we should also have noticed Colombier's excellent 'Code de Médecine Militaire,' in five volumes; Hecker's 'Manuel de Médecine pratique Militaire;' 'Revolat's 'Nouvelle Hygiène Militaire,' in one volume, published in 1803; and the valuable article on that subject by Vaidy, in the 'Dictionnaire des Sciences Médicales.'

The 'Military Medical Guide Books' may be divided into two classes those relating to the position of the army medical officer and to his *military* duties, and those which treat of the means of preserving the health and promoting the efficiency of soldiers: the study of the first will conduce much to his own comfort, and materially aid him in his duty to his superiors and his brother officers; while the study of the second will teach him how to alleviate many of the hardships incident to military life, and to increase the comfort, and improve the condition, moral and physical, of those intrusted to his care.

I. In the first class may be included Dorat's and Puel's Manuals; but we shall not enter into a detailed consideration of these works, as the regulations of the medical department of the French army differ very much from our own. We would, however, strongly recommend Puel as a model for a similar manual for medical officers of the British army. It consists of four parts. The first treats of the organization of the department and the composition of the corps of army medical officers: in this are included the regulations regarding appointments, promotions, pay, uniform, subordination, &c. The second part comprehends the duties of medical officers in regiments, and in hospitals at home, including the examination of recruits and invalids: and the third the duties of medical officers in regiments, in hospitals, and in field hospitals with an army on active service. The fourth part contains the laws regulating, 1st, the recruiting of the army; 2d, the condition of the officers of the army as regards their rank, their position, (viz. full-pay, half-pay, and retirement with or without pension,) their pay, &c.; 3d, the pensions of the army; and 4th, the king's warrant regulating courts of inquiry. It also contains tables of the pay, allowances, and pension of the different ranks of medical officers, forms of the various reports and certificates required by the regulations, and a pharmaceutical formulary for the use of military hospitals.

The 'Aide-Mémoire' by Maillot and Puel, besides the topics just enumerated, contains full instructions regarding the medico-legal questions which may be submitted to army medical officers. These relate chiefly to wounds, their nature and severity,—have they been inflicted during life? are they homicidal, suicidal, or accidental? to drowning, hanging and strangulation, and to rape. There is also a section on the adulterations to which the soldier's food is liable, whether from accident, or fraud on the part of the contractor, with the best means of detecting them Examples are given of the reports required in these various cases, which cannot fail to prove most useful to the medical officers. We do not intend at present to enter into a consideration of these questions, but will reserve our observations for some other time. The work has been published under the sanction of the war minister, and may therefore be looked on as demi-official, which of course enhances its importance.

The little work by Sarlandière holds a position between those already noticed and the treatises on military hygiene In the introduction he says he has collected together, in as small a space as possible, the most necessary information on the following subjects. 1. All that concerns military surgeons in the various situations in which they may be placed, whether as regard their position, or their duties in regiments, ambulances, and hospitals. 2. Everything relating to the maintenance of the health of the soldier, or a summary of military hygiene. 3. The symptoms, causes, and treatment of the diseases which may be treated without sending the soldier into the hospital. 4. The operations which should be performed on the field of battle, and the application of the first dressings. 5. A pharmacopœia for the use of the surgeons of regiments and field hospitals. The appendix contains an enumeration of the diseases and disabilities which exempt recruits from military service, and give rise to the invaliding of soldiers, with extracts from the regulations, laws, and decrees, or warrants relating to the medical department of the army. This work contains many very excellent remarks and much useful information, but from a desire to render it what it professes to be, a vade-mecum, it has been so condensed that it must rather be regarded as an aide-memoire to the experienced officer, than as a manual and guide to the young one who has just entered the service and has still to learn his duty.

II. The second class of these Guide Books includes such works as relate to the maintenance and improvement of the health and efficiency of soldiers. Of late years the attention of the authorities in this country has been much directed to the state of public health, particularly in towns, and commissioners have been appointed to inquire into the subject, with a view to legislative enactments being founded on their reports. But, partly from want of experience, and partly from circumstances which it will not be easy to control, partial difficulties have arisen in carrying out the various schemes, from which, unless some remedy is devised, most serious consequences may result. To illustrate this by an example, we would refer to the metropolitan improvements at present being carried on under the superintendence of the Commissioners of Woods and Forests. One of the great advantages promised from these alterations was that the new lines of streets were to be carried through the midst of a most thickly populated part of the metropolis—including the Rookery and the Seven Dials—which would thereby be opened up, the ventilation be much improved, and the standard of health in the neighbourhood raised. Now, it seems highly probable that these desirable ends will be accomplished, but let us examine in what manner. The houses in progress of erection in the new streets, are of a very different class from those which have been pulled down, and as regards rent and the nature of the accommodations, are quite unsuited for the ejected tenants. A great migration has consequently taken place, principally we believe to the already densely-crowded courts and alleys of Drury lane and of Westminster, where this influx of ill-fed and unfortunate beings can scarcely fail to produce typhus or some other disastrous epidemic in the ill-ventilated, dirty, over-crowded cellars and hovels, into which they are crammed. Before the contemplated improvements in Westminster are carried into effect, it is surely the duty of Government to see that adequate provision is made for the domestication of those who are thus turned adrift without any one to care for them. We would earnestly recommend the Health of Towns Commission, in any measures they may bring forward, to point out the necessity of providing suitable dwellings for the poor before pulling down their present ones, however miserable they may be. Unless some such system is adopted, we shall have the repetition of a scene, by no means uncommon in this country—large sums of money expended in alleviating the misery of the poor, and arresting the spread of contagious disease, the existence of which might have been prevented by a little foresight and a moderate but well-timed outlay. Let not the wealthy suppose this a question that does not affect them; when typhus becomes epidemic it is impossible to say where it will stop, and they may have the melancholy lesson brought home to them, as has repeatedly happened in the epidemics which have prevailed in Glasgow, of the fatal effects arising from the neglect of the principles of hygiene.

These are difficulties, however, which do not attend the adoption of hygienic measures in the army; as the Government is bound to feed, clothe, and house the soldier, so before any extensive alteration is carried out, provision is made that he may not suffer in consequence. Motives of policy, however, occasionally render it necessary to place him in an unhealthy locality, but many of the injurious effects of this may be averted by the adoption of judicious sanitary precautions. These have unfortunately not always been sufficiently attended to; but we may notice it as one of the valuable practical results of the Army Statistical Reports, that many measures have been adopted in compliance with the suggestions contained in them, and with very satisfactory consequences. That we may not be supposed to attach *undue* importance to the benefits arising from attention to military hygiene we may refer to a few instances in which marked advantage has been derived from it. As regards the position of barracks, a striking example is afforded in the diminution of fever which followed the removal of the Augusta Arsenal, United States, from the Savannah to the Sand-hills, three miles distant.* The benefit derived from quartering the white troops in Jamaica, at Maroon Town, and Newcastle, where the mortality averages from two to three per cent., instead of at Port Antonio, Montego Bay, and Up Park Camp, where it exceeded fourteen per cent., is too obvious to require comment. The reduction in the prevalence and fatal character of dysentery, subsequent to the issue of

* Br. and For. Med. Rev., July, 1843, p. 41.

mattresses stuffed with coir (the husk of the cocoa nut,) and a blanket, to the soldier in Ceylon,* and the same result from the diminution in the issue of salt meat in the West Indies, and on the coast of Africa, are worthy of remark. The only other instance we will at present notice was the prevalence of colica pictonum in the United States army prior to 1830, and its complete cessation after the use of white lead for cleansing their belts and gloves had been interdicted, and pipeclay substituted.†

While soldiers are equally subject to topographical and atmospherical influences with persons in civil life, there are other agencies in regard to which they are differently situated. The principal of these are barracks, clothing, diet, duty, exercise, amusements, education, and punishments. In these various particulars (punishments excepted) civilians may consult their convenience or please their fancy, if their purse permits; but there are certain rules laid down with which the soldier must comply. It is, consequently, of the highest importance, in framing these rules, that great care should be taken that they are in no way likely to injure his health or unnecessarily interfere with his comfort; and it is the duty of the military medical officer to see that this is the case, or to point out to his commanding officer, or the head of his department, in what respects he considers them injurious, and the most suitable means of remedying them.

We propose, at present briefly, to advert to the various topics enumerated above, and to point out the principal conditions to be fulfilled as regards each of them.

1. *Barracks.* Hitherto, in selecting a site for barracks it has been customary to consult only the officer commanding in the district, station, or colony, and the engineer department. They have in consequence been usually built in a position whose recommendation chiefly or wholly consisted in its military capabilities, while the question of its salubrity was little thought of. The result of this system has been a fearful sacrifice of life in many of our colonies, and necessarily a proportionate waste of money. Had the White troops of Up Park Camp, Jamaica, during the twenty years included in the Military Reports, been quartered at Newcastle in the Blue Mountains, a change which has been recently carried out, upwards of £50,000 would have been saved during that period, estimating each soldier to cost government £30 before he is equipped, drilled, and landed in that island—an estimate which, we believe, does not exceed the actual outlay. Leaving humanity, then, altogether, out of the question, and the *right* which the soldier has to be protected from *unnecessary* risk of life and deterioration of constitution in serving his country, and viewing it in the extremely commercial light of £. *s. d.*, it is obviously the interest as well as the duty of government to quarter the troops in the most healthy places that can be found, so long as these are compatible with the requirements of the service. A heavy expense having been incurred in building a barrack, it would be extremely difficult to persuade the authorities to abandon it and build another on the plea of the site being insalubrious, but we trust

* British and Foreign Medical Review, April 1844, p. 336.

† An instance of the value of attention to hygiene in the navy will be found in the case of H. M. ship Valorous, as quoted from Dr. Combe's 'Principles of Physiology,' in the 1st vol. of this Review, p. 334.

that in future measures will be adopted for obtaining information on this vitally important point before any permanent station for troops is established. Where doubts are entertained on the subject, wooden or iron barracks, similar to the houses now sent out to the colonies for emigrants might be employed, as they could be moved at a trifling expense if the situation proved unfavorable. Mutel thus sums up the conditions requisite for a healthy barrack: "It should be raised at least four feet above the ground, situated at a sufficient distance from rivers, and more especially from marshes, ponds, muddy streets, common sewers, pits for steeping hemp, the neighbourhood of cemeteries, slaughter-houses, lime-kilns, and in a word from all establishments for manufactures of an insalubrious nature. There should be a free circulation of air round the building, which preserves it from damp, and it ought to be entirely isolated from other buildings, and have an eastern exposure." We may add that the ground on which it stands should be thoroughly drained.

The two most important points to be attended to in a barrack are, to ventilate the rooms properly, and to avoid over-crowding. The subject of ventilation has, till of late years, been grievously neglected, and we consequently yet stand in need of practical experience as to the best modes of conducting it. We trust that the works of Dr. Reid and others will meet with the attention they deserve from the corps of Royal Engineers, and that these officers will be more successful than they have hitherto been in affecting so desirable an improvement. Whatever means are adopted, they ought to be of such a kind as to be out of the control of the private soldier; a non-commissioned officer should be intrusted with the regulation of the apparatus, and be held responsible for the room being at all times properly ventilated. This remark is applicable to guard-rooms also, their ventilation being generally very defective.

The second object will be best effected by having rooms of moderate size, and by care on the part of the barrack department to allot them, not according to the superficial extent of flooring, but according to their cubic contents. Sarlandière states that the space allowed to each man ought not to be less than 1307 cubic feet (cinq toises cubes,) and Mutel that it should be at least 424 (douze métres cubes.) Motard, in his 'Essai d'Hygiène générale,' recommends 866 to 1039 cubic feet for each patient in hospital; the prison inspectors allow 1000 feet for each prisoner; and Tredgold, in his work on Ventilation, considers 600 cubic feet to be necessary for the preservation of health. We are not so sanguine as to expect that the space recommended by Sarlandière will ever be given to the soldier in barracks, though it would doubtless be a most beneficial measure, but we do trust the day is not far distant when Tredgold's estimate will be adopted as the *minimum*, and strict attention to it enforced. A considerable amount of disease among soldiers is, we fear, too justly attributable to their constantly breathing the vitiated atmosphere of over-crowded and badly-ventilated barrack-rooms.

2. *Clothing*. Much has been written on the subject of the soldier's clothing, and alterations without end have been suggested. We do not agree with those who would sacrifice appearance altogether, to the plea of convenience, or the *unproved* assertion of the dress being injurious to health. A great moral advantage is gained by getting a soldier to take a pride in his personal appearance, and we would willingly make some sacrifice to effect this. The only very objectionable parts of his dress are the stiff leather stock and the chako. For the former a black silk or muslin one might be advantageously substituted; the chako is too heavy, from its shape compresses the head very much, and affords no protection from the rain. Mutel recommends instead of it, a helmet of glazed leather, which we believe has been recently adopted in the Prussian army. The new chakos lately issued to some of our regiments are said to be much less objectionable than the old ones, but it is a pity they are not a little more ornamental.

A most important addition to the dress of the soldier would be a flannel under-waistcoat. When dismissed from parade or drill, or after a march, it is impossible to prevent him throwing off his coat to cool himself; the perspiration is often suddenly checked, and catarrh, pneumonia, or pleurisy, the result. This would be much less likely to occur, were the chest covered with flannel, and in winter it would likewise protect him from the effects of cold when on duty. Mutel objects to the use of flannels, because "they become at once a cause of disease, by rendering the skin more sensitive, and a powerful obstacle to their cure (diseases of the chest and rheumatism,) by depriving us of a means of treatment; and when the soldier once becomes accustomed to their use, it is dangerous to give them up." To this however we may reply that it is better to prevent than to cure disease. The pamphlet by Mr. Roberts on the clothing of soldiers contains many sensible remarks, and may prove useful in directing the attention of those in authority to the defects of the present system and the appropriate remedies. In stating, however, "that the defective clothing of the soldier is the chief cause of his ill-health," we think he attaches too little importance to other causes of disease. We are pleased to find the subject attracting attention, because if it leads to any investigation on the part of the military authorities, benefit to the soldier will probably result in the substitution of materials of better and more durable texture, and in an improved system of making-up and fitting the clothing. If Mr. Roberts continues his observations, as he promises to do, and we trust he will, a little more care would be well bestowed on the literary part of his labours. It is strange, as we have often remarked, that medical writers are so very careless on this point.

The great weight soldiers are obliged to carry, under the title of "regimental necessaries," amounting to about 40lbs., is very objectionable, especially when it is necessary to make long marches; the knapsack in which they are contained is also of a very bad construction, preventing, by its breaststraps, the due expansion of the chest at the very time it ought to be most free, when the soldier is in active motion, and respiration is consequently accelerated. Any alteration tending to lighten the weight of the contents of the knapsack, or any invention by which it could be made to sit well upon the shoulder without breaststraps, would be a great boon to the army. It can scarcely be doubted that the present mode of wearing it must exert an injurious influence on the health of the soldier, particularly if he is predisposed to pulmonary disease.

3. *Diet.* The diet of the soldier is amply sufficient to maintain him in health, but it might with advantage be a little more varied. The introduction of an evening meal into the army has been frequently advocated, but there are several obstacles to it, the principal one being the difficulty of providing the necessary sum out of the very small amount of surplus pay at the disposal of the soldier, probably not averaging above 3*d.* or 4*d.* per day, and which he naturally wishes to expend for his own pleasure. It might perhaps be advantageous to make four o'clock the dinner hour instead of one, as the period between meals—at present nineteen—would be thereby shortened. The medical officer should frequently inspect the messes, when served out to the men, to ascertain that the provisions are of good quality and sufficiently cooked.

4. *Duty.* It is a desirable object at all times to reduce the amount of night duty as much as possible ; soldiers ought to have at least four nights in bed, as when they have less, for any length of time, it cannot fail to tell upon their health. It has been argued that when the duty is severe, they have not time to run into dissipation, and the consequent sickness and crime are thereby prevented. This, if correct, would only be applicable to day duty ; but it is possible so to harass them by over work as to make them rush into those very excesses we desire to prevent. At all events it would be much better to try the influence of moral in means, education for instance, in restraining crime, than physical. Care should be taken to protect the men when on sentry as much as possible from the vicissitudes and inclemency of the weather.

5. *Exercise.* Under this head may be included parades, drills, and marches. As the amount of these varies much in different regiments, and under different circumstances, we may merely remark that it is the duty of the medical officer to watch carefully that they are not carried on in such a manner, or to such an extent, as to be injurious to the health of the men. They ought not to be continued too long at a time, should be varied as much as possible, and in summer, or in warm climates, care should be taken to get them over if practicable before the heat of the sun becomes oppressive. These remarks are still more applicable to the case of recruits who have just joined, than to soldiers who have acquired a knowledge of their duty and become habituated to the service. In recommending that soldiers should be accustomed to active exercise, Sarlandière remarks, "I am far from approving of that *perpetual monotony of drill* to which some commanding officers subject their men ; for by keeping them always at the same exercise they are soon wearied, and become disgusted with their condition." On these points much of the comfort and health of the soldier depends, but they must necessarily be left in a great measure to the discretion of the commanding officer ; when, however, they affect the health, it is the surgeon's duty to point this out, and firmly, but respectfully, to remonstrate on the subject.

6. *Amusements.* The beneficial effects of athletic games upon the health has been acknowledged from the earliest periods, but it is only of late that Government has taken any steps to encourage them in our army. In compliance with the recommendation of the Military Punishment Commission, fives-courts have been built, and cricket grounds provided in many of the barracks and garrisons throughout the kingdom. This cannot fail to be of great advantage, by affording an agreeable amusement to the men when off duty, by furnishing them with the means of healthy exercise, and thus keeping them out of the canteen and other places of low debauchery ; but to derive the full benefit from any measures of this nature, the officers must take an interest in, and endeavour to promote them. In addition to the games mentioned above, we would recommend the adoption of football, quoits, skittles, foot races, and others of a similar nature. These have at different periods been introduced into several regiments, and promoted by trifling subscriptions from the officers; and where they have afterwards been discontinued, it has arisen from want of proper management, or from indifference on the part of those in authority. Were a system of this kind more generally pursued, and the men encouraged to distinguish themselves by some occasional trivial indulgence, one of the most unpleasant duties of a commanding officer, that of punishing delinquencies, would be materially lightened, and the health and efficiency of the men improved.

7. *Education.* While we are fully impressed with the advantages to be derived from the introduction of such amusements, in promoting the health and efficiency of the soldier, and in diminishing the amount of crime, we anticipate still greater benefit from the adoption of a general system of education in the army. Soldiers ought to be considered as something more than mere automata, and while they are taught their military duties and have the necessity for an implicit and unquestioning obedience of orders impressed upon them and enforced, adequate means should be adopted to cultivate their intellectual faculties, and thereby afford them a rational means of employing their leisure hours, and an opportunity of qualifying themselves to occupy a respectable position in society when discharged from the military service. To effect this desirable object no recruit should be dismissed drill till he is reported able to read, care being taken that he understands what he reads, to write legibly, and to be acquainted with the first rules of arithmetic. Were this done, colonels would have a much better opportunity of selecting non-commissioned officers, and soldiers would be induced to spend part of their leisure time in reading instead of in the canteen; and when at length discharged for disability, or length of service, would be better enabled to provide for themselves and families, and would reflect credit on the service to which they had belonged.

We infer that this subject has received more attention in France than in England. Mutel, in treating of education, says, "the nation which teaches its young soldiers—born of poor parents who have been unable to give them the benefit of education—reading, writing, grammar, history, geography, arithmetic, drawing, and music, has not a bad government, since it knows how to recompence those who commence their career of citizens by sacrificing to the defence of their country the first-fruits of their youth." We much doubt whether the French actually teach these various branches, but the profession of doing so is an acknowledgment of the duty; and although we do not expect our Government to undertake so much, we sincerely desire to see every soldier receive a plain useful education. Reading-rooms having already been established, and a sum of money (small enough, it is true,) being annually expended for books for the soldiers' libraries, it is surely but reasonable to expect that steps should be taken to render them available to all.

But while the intellectual education of soldiers is neglected, we fear the provision for their moral and religious instruction is even more deficient. Something more is required than mere attendance at divine service, or hearing the liturgy read once a week: to render a clergyman's ministrations useful, he must mingle with his flock, must become acquainted with their habits, their wants, their virtues and vices, and must watch for opportunities of addressing to them a word in season. The recently appointed chaplain-general to the forces, the clever author of the 'Subaltern,' must, from his early career, have a practical knowledge on these subjects, which peculiarly qualifies for the superintendence of the department; and we hope soon to learn that he is directing his talents, and using his influence with those in power, to secure an adequate provision for the spiritual wants of the army in all quarters of the globe.

8. *Punishments.* The consideration of the influence of punishments on health is one of very great importance, but to which our space will not do more than permit us briefly to advert.

The principal punishments inflicted by sentence of a court martial are flogging and imprisonment: the former of these, as at present administered, is not likely to injure the health;* would that we could say the same of the latter! The diet in many of the prisons, though adequate enough for the unfortunate civil prisoners, who probably prior to their entrance rarely knew what it was to have a full meal of nutritious food, is by no means sufficient for the soldier, who has been accustomed to his $\frac{3}{4}$lb. of meat, with soup, potatoes, bread, and coffee daily. This is no theoretical objection, but has been forced on our conviction, by having frequently seen men return to their regiments very much debilitated even after short periods of imprisonment, and by a few cases in which after a long period (twelve months) they scarcely performed another day's duty, and ultimately were discharged the service or died of pulmonary consumption. So obviously was this a cause of disease in the Penitentiary at Millbank, that a few years ago, a portion of that building was appropriated exclusively to soldiers, and a different scale of diet adopted for them; a system which should have been extended to all the prisons in the kingdom. As military prisons are now to be generally established, and offenders sentenced by courts marshal no longer to be sent to the civil gaols, we trust the subject of their diet while so confined, will receive the attention it deserves, and that care will be taken to prevent a definite punishment being converted into a very severe and indefinite one, by entailing on the individual years perhaps of misery and suffering from ruined health, or an early grave. We must protest against punishing soldiers by interference with their food, as in the case of imprisonment with bread and water, because it is an infliction of a very unjust and unequal character, and the ultimate effects of which cannot be appreciated.†

* It would be out of place here to discuss the question of the abolition of corporal punishment, but we may ask those who raise an outcry against it as being degrading to the soldier, and recommend secondary punishments, i. e. imprisonment, as a substitute for it, whether is it more degrading to a soldier to be flogged or to be associated with convicted felons and hardened gaol birds, and whether is he more likely to be improved by castigation and the fortnight or three weeks he has for reflection in hospital afterwards, or by the acquaintances he will form in prison among the worst and most abandoned characters in the kingdom? Most willingly would we see flogging abolished, for it is one of the medical officer's most disagreeable and responsible duties to superintend it; but we fear, constituted as our army is, its continuance is a matter of absolute necessity.

† To those who feel an interest in this subject, we would strongly recommend a perusal of a highly interesting series of papers in the 'United Service Magazine' for 1843-4, by Henry Marshall, Esq., Deputy-Inspector-General of Hospitals, entitled 'Historical Notes on Military Punishments.'

The minor punishments ordered by commanding officers are not likely to prove detrimental unless when carried to excess, if we except drill in heavy marching order, the objection to which will be found in the remarks we have already made on the soldier's knapsack.

We have said nothing on the important subject of personal cleanliness, because that is already well enforced throughout the army, so far at least as regards the face, hands and feet, and the dress. We hope, however, the popular movement now going on to provide baths for the working classes will suggest to the authorities the propriety of building baths in every barrack, thereby affording to the soldier the means of general ablution which at present are quite out of his reach: where space can be procured large swimming baths ought to be erected and the men be instructed in swimming, an acquirement which might be of the utmost consequence to them on service.

To improve the hygienic condition of the soldier must be a work of time, and the medical officer will meet with numerous obstructions in his endeavours to effect this laudable object. There will be many prejudices to overcome, and he may expect to have his plans sneered at, or neglected, and occasionally his motives misconstrued. But let him not weary in well doing; *gutta cavat lapidem non vi sed sæpe cadendo;* and it requires a good deal of dropping to wear through the prejudices of the heads of departments when no precedent can be adduced, or where an outlay of money is required. We have heard suggestions for increasing the comforts of soldiers objected to by officers of high standing, because they said "by coddling the men" you would make them effeminate, and when it became necessary to take the field, they would be unfit to go through the hardships of a campaign. We have no fear of such a result; the greater the efficiency and the higher the standard of health of the soldier, the better will he be able to endure the fatigues of active service; and when he finds every attention paid to his comfort in time of peace, he will the more cheerfully submit to privations when they unavoidably occur. Notwithstanding all that has been adduced in favour of cold, hunger, and discomfort, in fortifying a constitution against the contingencies of famine and fatigue, the practice deserves no commendation; privation of food and insufficient clothing stunt the growth, and diminish the powers of the body, at the same time that they impair the energies of the mind.

The works of Sarlandière and Mutel are evidently the productions of men of much experience, who have seen a good deal of service and have the interests of the soldier at heart. The latter goes more into detail, and is therefore perhaps preferable as a guide book, but both are well deserving of attentive perusal. They are very practical and are tolerably free of that inflated style of writing for which modern French works are so remarkable. We would have been better pleased, however, had Mutel acknowledged the sources from which he derived his information: he scarcely in any instance quotes his authority; and although he appears to have drawn liberally from Maillot and Puel's Aide-Memoire, we do not find their names mentioned throughout the work.

